# Harnessing AI for Improved Detection and Classification of Pleural Effusion: Insights and Innovations

**DOI:** 10.1155/carj/2882255

**Published:** 2025-08-06

**Authors:** Geran Maule, Ahmad Alomari, Abdallah Rayyan, Ogbeide Aghahowa, Mohammad Khraisat, Luis Javier

**Affiliations:** ^1^Department of Clinical Sciences, University of Central Florida College of Medicine, Orlando, Florida, USA; ^2^Department of Graduate Medical Education, HCA Florida North Florida Hospital, Gainesville, Florida, USA; ^3^Department of Medicine, University of Benin School of Medicine, Lagos, Nigeria

## Abstract

The detection and classification of pleural effusion present significant challenges in clinical practice, often contributing to delayed diagnoses and suboptimal patient outcomes. Recent advancements in artificial intelligence (AI) and machine learning (ML) techniques hold substantial promise for enhancing the accuracy and efficiency of pleural effusion diagnostics. This paper reviews the current landscape of AI applications in pleural effusion detection, synthesizing findings across diverse studies to illustrate the transformative potential of these technologies. We examine various ML models, including deep learning and ensemble methods, that leverage clinical, laboratory, and imaging data to improve diagnostic performance. Notably, models such as Light Gradient Boosting Machine (LGB) and XGBoost have achieved accuracy levels up to 96% and high AUC values (e.g., AUC = 0.883 for pleural effusion differentiation). This overview highlights the importance of integrating diverse diagnostic parameters to enhance pleural effusion diagnostic accuracy and outlines future research directions essential for optimizing patient management and outcomes.

## 1. Introduction

Pleural effusion, the accumulation of fluid in the pleural space, represents a prevalent clinical challenge encountered across various medical specialties. Common etiologies include heart failure, malignancies, and infections, necessitating timely and accurate diagnosis to guide effective management strategies. Traditional diagnostic methods rely on a combination of clinical evaluation, imaging studies, and laboratory analyses of pleural fluid. However, these approaches often entail time-intensive procedures and are prone to misdiagnosis, partly due to the subtle clinical and imaging features of pleural effusions [1].

The rise of artificial intelligence (AI) and machine learning (ML) offers a novel avenue to enhance pleural effusion diagnosis by analyzing complex data patterns beyond human capability. Various ML models have demonstrated efficacy in classifying pleural effusion types and predicting underlying causes. Studies show that ML algorithms, particularly ensemble and deep learning (DL) methods, improve diagnostic accuracy by integrating clinical and imaging data. However, the practical implementation of these models presents challenges, such as the need for high-quality, large-scale data, and seamless integration with existing clinical systems.

This paper explores recent advancements in AI-driven approaches for detecting pleural effusion, synthesizing insights across studies. By examining innovative methodologies, we aim to underscore the potential of ML technologies to revolutionize pleural effusion diagnostics and identify key factors influencing real-world clinical adoption.

## 2. Methodology

### 2.1. Literature Search Strategy

We conducted a systematic search across multiple databases, including PubMed, IEEE Xplore, and Google Scholar, covering publications up to July 25, 2024. The search aimed to capture a comprehensive range of studies on ML applications in pleural effusion detection. Included studies were original research articles that employed ML models for pleural effusion diagnosis, classification, or characterization. Exclusion criteria were applied to exclude review articles, studies with insufficient data, or those focused on conditions unrelated to pleural effusion.

### 2.2. Data Synthesis and Analysis

A narrative synthesis approach was employed to organize findings, categorizing studies based on ML model types (e.g., DL and ensemble methods) and data types (e.g., clinical and imaging). This framework enabled a comparative analysis of performance metrics, such as accuracy, sensitivity, specificity, and AUC values, providing a structured overview of each model's strengths and limitations.

## 3. Results

The application of ML and DL in pleural effusion diagnostics demonstrates significant advancements across various studies, each contributing uniquely to model development, diagnostic accuracy, and clinical relevance. This section provides a comprehensive study-by-study breakdown (Table 1), highlighting key findings and comparative performance metrics.

1. *Kim et al. (Light Gradient Boosting Machine [LGB] Model for Multitype Pleural Effusion Classification)*
  Kim et al. developed a model using LGB to classify pleural effusion types—transudative, malignant, parapneumonic, tuberculous, and other effusion types. This retrospective study, which included data from over 2000 patients, utilized clinical and laboratory data to train five different classifiers, among which the LGB model achieved the highest accuracy, with an AUC of 0.930 in the validation set and 0.916 in the extra-validation set, the LGB model demonstrated that integrating key biomarkers (e.g., pleural lactate dehydrogenase, protein, and adenosine deaminase) could effectively support differential diagnosis of pleural effusion etiologies. Notably, feature selection showed that a reduced set of 18 features provided accuracy nearly equivalent to the full feature set, enhancing model efficiency.
2. *Wei et al. (XGBoost for Malignant Pleural Effusion Identification)*
  Focusing specifically on malignant pleural effusion, Wei et al. employed the XGBoost algorithm, using routine laboratory data to distinguish between malignant and benign effusions. The model outperformed traditional diagnostic markers such as carcinoembryonic antigen (CEA) in both specificity and sensitivity. Across training, validation, and testing cohorts, XGBoost achieved consistently high AUCs (0.903, 0.918, and 0.886), underscoring the value of common laboratory biomarkers in malignant pleural effusion detection. These results highlight the utility of XGBoost as a practical model for routine clinical settings where advanced imaging may be unavailable.
3. *Zhang et al. (Tumor Marker Integration for Malignant Pleural Effusion Detection)*
  Zhang et al. evaluated the diagnostic performance of various ML models using combinations of tumor markers (CEA, CA125, CA153, and CA19-9). Using XGBoost, they found that combining CEA and CA153 provided the highest accuracy for malignant pleural effusion diagnosis, achieving an AUC of 0.921, with 85% sensitivity and 98% specificity. This study reinforced the potential for multimarker diagnostic models to surpass single-marker approaches, particularly in cases where malignancy must be detected quickly and accurately.
4. *Lee et al. (Contrastive-Loss Model for Pleural Effusion Etiology Classification)*
  Lee et al. applied a contrastive-loss DL model to classify pleural effusion etiologies, achieving a micro-AUROC of 0.942 in the validation set and 0.867 in the extra-validation set. This model excelled by creating an embedding space that differentiated typical and atypical disease cases, using methods like UMAP and t-SNE for visualization. The approach allowed for intuitive interpretation of model outputs, supporting clinicians in distinguishing between similar pleural effusion types through visualized patterns of true and false positives.
5. *Widodo et al. (Support Vector Machine (SVM) for Imaging-Based Pleural Effusion Detection)*
  Widodo et al. explored the application of SVM in detecting pleural effusion from chest radiographs, achieving an impressive 96% accuracy and 100% precision. By focusing on morphological features in chest images, the study demonstrated the potential for SVM to serve as a rapid, reliable diagnostic tool in emergency or resource-limited settings where access to lab data might be constrained.
6. *Wang et al. (3D/2D U-Net for CT-Based Pleural Effusion Segmentation)*
  Wang et al. developed a segmentation and classification model using a combination of 3D and 2D U-Net models on thoracic CT images. The model achieved an AUC of 0.842 for distinguishing malignant from benign effusions, with high segmentation accuracy validated by experienced radiologists. The model's ability to outline pleural effusion areas in 3D provided a comprehensive view, potentially aiding in clinical settings where precise localization is essential for treatment planning.
7. *Nose et al. (Noninvasive Bioelectrical Impedance Analysis for Pleural Effusion Estimation)*
  Nose et al. leveraged LGB in a noninvasive setting, using multifrequency bioelectrical impedance data to estimate pleural effusion presence among heart failure patients. The model achieved an AUC of 0.905, offering a portable, noninvasive alternative for pleural effusion diagnosis in settings where imaging may be impractical. This method demonstrated that combining clinical and impedance data can produce accurate diagnostics for continuous monitoring.
8. *Vukovic et al. (DL for lung ultrasound [LUS]–Based Pleural Effusion Segmentation)*
  Using DL to segment pleural effusion in LUS images, Vukovic et al. achieved a Dice Similarity Coefficient (DSC) of 0.70. Their model provided segmentation results comparable to those of experienced clinicians, highlighting its potential to support bedside pleural effusion volume estimation and facilitate safer thoracentesis procedures by precisely locating effusions for needle guidance.

## 4. Main Text

The application of ML in diagnosing pleural effusions represents a paradigm shift in clinical diagnostics, with promising advancements in accuracy, efficiency, and patient outcomes. However, transitioning these findings from research to practice presents several challenges and opportunities.

The models in the reviewed studies demonstrate high accuracy in specific diagnostic scenarios. For instance, Kim et al.'s LGB model accurately classified pleural effusion subtypes using clinical and lab data [1] and Wei et al.'s SVM model excelled in rapid binary classification of pleural effusion in emergency imaging scenarios [2]. Such diversity suggests that a single, one-size-fits-all ML model may not be feasible. Instead, model selection may need to be tailored to clinical contexts, with imaging-based models like SVM or U-Net better suited to acute, imaging-heavy environments, and lab-data-focused models ideal for differential diagnosis in broader clinical settings. Future research should explore ensemble methods that integrate both lab and imaging data to create more comprehensive, adaptable diagnostic tools.

For ML models to gain clinician acceptance, they must be interpretable. Studies like Lee et al.'s contrastive-loss model provide valuable insights by visualizing the embedding space, aiding clinicians in distinguishing between typical and atypical cases [3]. Similarly, Zhang et al.'s tumor marker-based XGBoost model offers interpretability by highlighting key markers like CEA and CA153 [4]. Clinician trust is often rooted in understanding a model's decision-making process, which suggests that enhancing model transparency through explainable AI (XAI) methods is crucial. The next steps should focus on integrating XAI techniques to clarify ML models' internal mechanics, allowing clinicians to see and verify how models prioritize specific features in their assessments [5].

Despite the potential of ML to standardize and improve diagnostics, these models are only as robust as the data they are trained on. Many reviewed studies used data from localized, often homogeneous patient populations, potentially limiting the models' applicability across diverse demographics. This could lead to biased predictions if models are applied in populations they were not trained on. For example, certain tumor markers and clinical characteristics may vary by ethnicity or socioeconomic factors, influencing diagnostic accuracy [6]. It is crucial for future studies to validate models across multicenter and multiethnic datasets to ensure these tools are equitable and broadly applicable.

Practical integration of ML tools into clinical settings faces logistical challenges, especially concerning computational power and model updating [7]. Nose et al.'s bioelectrical impedance model, which uses portable, noninvasive data, exemplifies a solution tailored for bedside or rural applications where imaging might be unavailable [8]. However, complex imaging-based models like U-Net may require high computational resources, potentially limiting their use in settings with limited infrastructure. Developing lightweight or cloud-based diagnostic tools could facilitate real-time assessments in diverse healthcare environments, particularly beneficial in resource-limited or emergency settings [9].

Several studies, such as those by Kim et al., suggest that combining diverse data sources (e.g., imaging, lab data, and bioimpedance) may enhance diagnostic accuracy [1, 10]. Multimodal models that integrate clinical, laboratory, and imaging data could provide a holistic approach to pleural effusion diagnosis, potentially outperforming single-modality models in complex cases. Prospective studies should explore multimodal ML frameworks, with a focus on validating these models across multiple institutions to ensure consistent performance. Additionally, interdisciplinary collaboration with clinicians during model development and validation could help tailor these tools to meet real-world needs effectively [11].

Integrating ML-based diagnostics requires not only model reliability but also a shift in clinician training [12]. As ML tools become embedded in workflows, training programs must adapt to include ML interpretation and troubleshooting skills. Just as clinicians interpret lab values or imaging studies, they will need to understand how to read and act on ML-based diagnostic outputs. Professional development courses or certifications could help bridge this gap, fostering clinician competence in ML-based diagnostics and enhancing collaboration between healthcare providers and AI developers [13].

## 5. Conclusions

In conclusion, ML and DL models offer remarkable potential to improve pleural effusion diagnostics. However, transitioning these tools from research to widespread clinical use will require advancements in model interpretability, data diversity, and ethical validation. Moreover, clinician training and workflow adaptation are critical to the successful integration of ML diagnostics. Through interdisciplinary collaboration and targeted research efforts, AI-driven diagnostics can become a cornerstone of equitable, efficient, and precise healthcare for pleural effusion management.

## Figures and Tables

**Table 1:** ML model comparison for pleural effusion diagnostics.

Study	ML model	Input data	Task type	Accuracy (%)	AUC	Sensitivity (%)	Specificity (%)
Kim et al.	LGB	Clinical + laboratory	Classification	94	0.94	91	89
Wei et al.	XGBoost	Routine biomarkers	Classification	92	0.88	90	87
Zhang et al.	XGBoost	Tumor markers	Classification	89	0.91	88	85
Widodo et al.	SVM	Chest radiographs	Detection	96	0.89	95	100
Wang et al.	3D/2D U-net	Thoracic CT imaging	Detection	88	0.883	85	82
Nose et al.	LGB	Bioelectrical impedance + clinical data	Detection	83	0.84	76	88
Vukovic et al.	CNN + spatial transformer network (STN)	Lung ultrasound images	Detection	N/A	N/A	N/A	N/A
Lee et al.	Contrastive-loss	Laboratory data	Classification	81.7	0.942	84.1	94.1

## Data Availability

Data sharing is not applicable to this article as no datasets were generated or analyzed during the current study.
